# Histopathological, Histomorphometrical, and Radiographical Evaluation of Injectable Glass-Ceramic-Chitosan Nanocomposite in Bone Reconstruction of Rat

**DOI:** 10.1155/2015/719574

**Published:** 2015-02-08

**Authors:** Maryam Seyedmajidi, Seyedmahmood Rabiee, Sina Haghanifar, Seyedkamal Seyedmajidi, Seyed Gholam ali Jorsaraei, Homayoun Alaghehmand, Naghmeh Jamaatlu, Ali Bijani

**Affiliations:** ^1^Dental Materials Research Center, School of Dentistry, Babol University of Medical Sciences, Babol, Iran; ^2^Department of Materials Engineering, School of Mechanic Engineering, Babol University of Technology, Babol, Iran; ^3^Oral & Maxillofacial Radiology Department, School of Dentistry, Babol University of Medical Sciences, Babol, Iran; ^4^Fatemeh Zahra Infertility and Reproductive Health Research Center, School of Medicine, Babol University of Medical Sciences, Babol, Iran; ^5^Operative Dentistry Department, School of Dentistry, Babol University of Medical Sciences, Babol, Iran; ^6^Department of Oral and Maxillofacial Pathology, School of Dentistry, Babol University of Medical Sciences, Babol, Iran; ^7^Non-communicable Pediatrics Disease Research Center, Babol University of Medical Sciences, Babol, Iran

## Abstract

*Background*. Bone defects following tumor resection and osteolysis due to bone lesions, periodontal tissue disorders, and bone reconstruction are challenges that surgeons face. Gass-ceramic-chitosan nanocomposite contains chitosan, a derivative of crustaceans' exoskeleton. *Methods*. Thirty-two 6–8-week-old male Wistar rats were chosen. One hole on each right and left tibia was made. The right tibia holes were filled with injectable glass-ceramic-chitosan nanocomposite, and the left tibia holes were left empty. After 7, 14, 28, and 60 days, histopathological, histomorphometrical, and radiographical assessments were performed. *Results*. Radiographic density on days 7 and 14 was significantly higher in the right tibias than in the left tibias. Trabecular bone thickness, which was higher in the right tibias, increased from day 7 to day 60 in both right and left tibias, although not significantly. *Conclusions*. Glass-ceramic-chitosan nanocomposite is suggested for use in bone repair in cases of bone loss. More histopathological, histomorphometrical, and radiographical assessments are also recommended.

## 1. Introduction

Bone defects may occur because of various systemic and dental disorders. The nature of bone consists of polymer composites and calcium phosphate nanoparticles. Different materials have been introduced as bone substitutes. Using allografts may be more favorable in some cases, but because of possible immune reaction and infection transmission their application is limited. Therefore, surgeons and dentists have considered synthetic bone substitutes and bone tissue engineering as alternatives. Newly synthesized composite has the following advantages.

*(1) Using Bioactive Glass-Ceramic Nanoparticles*. Bioactive glass-ceramics are an important class of bone substitute materials that have been considered because of their ability to directly link to bone tissue [1, 2]. Their nanoparticles increase surface area and increase the osteogenic potential of glass-ceramic [3, 4].

*(2) Using Chitosan*. Chitosan is the second most abundant natural polysaccharide after cellulose. This natural polymer is extracted from marine resources (crabs and shellfish in general) [5, 6]. It has unique features including biological compatibility, biodegradability, antimicrobial activity, wound healing and healing by 75%, antitumor effects, nontoxicity, and compatibility with genes [6–8].

*(3) Injectivity of Substance Alternatives*. Injection systems are highly regarded because they are the least invasive [9]. In many cases, there is a need for a system that allows cell suspension; due to a lack of solid material before injection, their usage is easily possible [10]. Because these substances take the form of the place into which they are injected, they can be used in all positions, even those with irregular shapes. However, the problem of cell adhesion and release of bioactive molecules through mixing is resolved before injection [11, 12].

Materials that are used for bone grafting can be divided into two main groups: natural and synthetic. Natural bone grafts using autogenous bone are the gold standard for bone repair and regeneration. Synthetic grafts are divided into four main groups: metallic implants such as titanium and its alloys; stainless steel and cobalt-chromium alloys; ceramics such as calcium phosphate, alumina, carbon, and glass ceramics; polymers such as poly(methyl methacrylate), poly(urethane), ultrahigh molecular weight polyethylene, silicon, and polylactide; and composites of the first three groups, such as calcium phosphate-ceramic coatings on metallic implants and polymer-ceramic composites. Many studies have demonstrated the osteoinduction properties of calcium phosphate biomaterials such as synthetic hydroxyapatite ceramic in dogs and coral-derived hydroxyapatite ceramic in dogs, monkeys, and baboons [13]. The aim of this study was to perform histopathological, histomorphometrical and radiographical assessments of injectable glass-ceramic-chitosan nanocomposite in reconstructing rat bone.

## 2. Materials and Methods

### 2.1. Material Preparation

#### 2.1.1. Synthesis of Calcium Phosphate Glass-Ceramic by* Sol-Gel* Method

**Figure 1:**
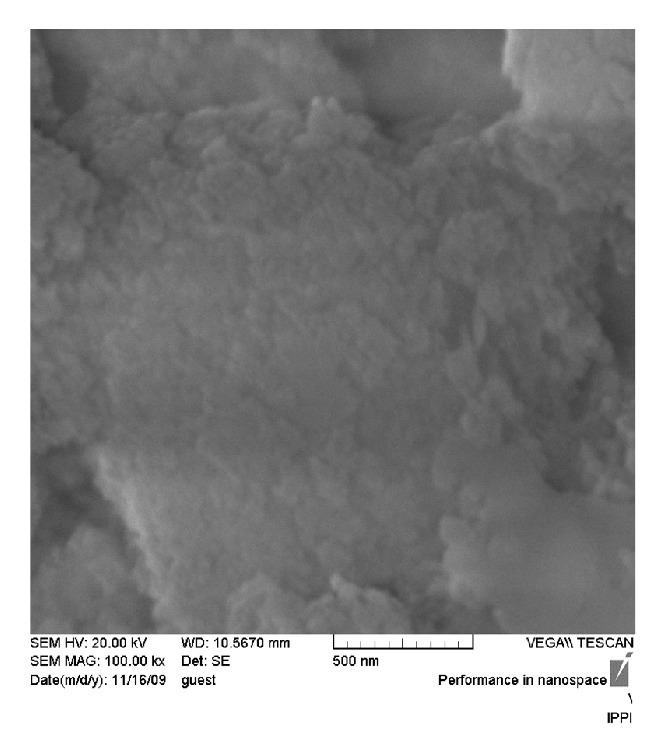
SEM picture of synthesized bioglass-ceramic and chitosan solution.

For the synthesis of calcium phosphate glass-ceramic by the* Sol*-*Gel* method, the following basic compounds were used: triethylphosphate and diammonium phosphate for providing P_2_O_5_ in the glass composition, calcium nitrate tetrahydrate as the CaO source, nitric acid and phosphoric acid to supply the sol environment, and tetraethyl orthosilicate as the SiO_2_ source. The prepared sol was stored in an isolated Teflon pan for 10 days until the condensation and the gel-forming process had started and the gel had formed. The created gel was given a special heat treatment in a dryer and an electric furnace to withdraw the gases and vapors, the crystallization process in the samples begins and scanning electron microscopy (SEM XL30) was used to characterize the morphology and grain size of samples. The samples were coated with gold before the examination. The crystal structure and the phase present in resulting samples were analyzed with X-ray diffraction (XRD). This instrument (Philips PW 3710) works with voltage and current settings of 30 kV and 35 mA, respectively, and uses Cu-K*α* radiation (1.540510 Å). SEM image of produced bioactive glass-ceramic and chitosan solution that could be used for study of the size, morphology, and homogeneity of samples are shown in Figure 1. As seen in figure, it is interesting to see that the particle size ranges in nano size. The XRD result of sample containing glass-ceramic and chitosan solution can be seen in Figure 1. In this figure, the pattern confirms the formation of the bioactive glass-ceramic.

#### 2.1.2. Providing an Optimal Chitosan Solution

At this stage a polymer solution was used to make the samples injectable. To prepare an injectable variety of bioglass ceramics, a chitosan solution was used and acetic acid was utilized as solvent. To create the best composite regarding injectivity and proper consistency, different percentages of chitosan in solvent were prepared.

#### 2.1.3. Glass Ceramic-Chitosan Nanocomposite

This phase included formation of the final composite. By mixing two presented components, the optimal concentration of powder nanoparticles and chitosan solution regarding injectivity and workability was obtained. Various amounts of glass-ceramic nanoparticles were added experimentally to varying amounts of chitosan solution, and then the best combination in terms of injectivity was selected. According to the results, if the ratio of 2% chitosan solution added to glass-ceramic powder is 2.1 mL/gr, it is suitable for clinical investigations in terms of engineering.

After the sample was completely isolated, it was sterilized in an autoclave for 15 minutes at a temperature of 120°C. The results showed that this sterile area did not change any properties of the composite.

### 2.2. *In Vivo* Experiments

This experimental study was performed on thirty-two 6–8-week-old male Wistar rats. Animals were anesthetized by thiopental odium injection, and the anesthesia was continued by diethyl ether. After shaving and disinfecting, the tibia was exposed by a lateral longitudinal incision. Two holes with a depth of 5 millimeters were created in both left and right tibias with a hand-held drill. Each tibia was drilled 1 cm lower than the knee joint with a round hand piece bur. The holes' sizes were the same and were equal to the bur diameter. The defects in the right tibias were filled by injectable glass-ceramic-chitosan nanocomposite, and those in the left tibias were left empty. Then, the muscle, subcutaneous tissue, and skin were closed in layers. Eight animals were sacrificed after 7, 14, 28, and 60 days in order, and the samples obtained were fixed in 10% formalin for one week.

### 2.3. Radiographical Analysis

Radiographic images were taken by PSP Soredex-Finland Digital Sensor under identical conditions of distance, kilovoltage peak (kVp), milliampere (mA), and time. The density of the defect was analyzed using the density option of Digora for Windows (V2.5) software. For this purpose, 50 Kv voltages and an 8 mA electric current for 0.2–0.4 seconds were used for radiographical imaging of the tibias. The distance of the tibias from the X-ray tube was set to 50 centimeters, and radiographic images were taken by Prostyle (Planmeca, Finland) phosphor plates sensors size 3 (occlusal) (PSP, Soredex-Finland). Then the phosphor plates were processed by PCT (Soredex-Finland) and analyzed by Digora for Windows (V2.5).

After images were processed, radiographic densities in different parts of the tibias were recorded, and the mean radiographic density of each tibia's defect was calculated and documented.

### 2.4. Histopathological and Histomorphometrical Studies

After radiographic imaging, the tissue surrounding the tibia was removed and the tibias were decalcified using 10% formic acid for 10 days. All sections prepared from each defect were photographed with an Olympus DP12 digital camera with microscopic ×40 magnification, and the images were analyzed by analySIS LS Starter software. The analySIS LS Starter software has the capability to measure distances and areas. By using this software the thickness of bone trabeculae can be calculated in histopathologic feature. Images were taken from the edge of the defect to the midsection and the appropriate images from the applied material area and not from the host bone were selected. Incidence of inflammation, foreign body reaction, and blood vessel counts in three microscopic fields, bone vitality, bone-biomaterials contact mode, the mean bone cells (osteoblasts, osteoclasts, and osteocyte) counts in three microscopic fields, trabecular bone thickness, new bone formation amount, and bone lacunae density in three microscopic fields were determined. The ranking of items was listed as follows.

 
*Inflammation*: The absence of inflammatory cells, Grade 0; scattered inflammatory cells (mild inflammation), Grade I; 5 to 10 inflammatory cells focally, Grade II; 11 to 50 inflammatory cells focally, Grade III; and >50 inflammatory cells focally, Grade IV.
 
*Foreign body reaction*: Presence of granulomatous reaction giant cells, +; absence of granulomatous reaction giant cells, −.
 
*Bone vitality*: Presence of osteocytes in trabecular bone lacunae, vital; absence of osteocytes in trabecular bone lacunae, nonvital.
 
*Trabecular bone thickness*: More than 60 microns (thick), Grade I; 20 to 60 microns (average), Grade II; and 1 to 20 microns, Grade III.
 
*Bone-biomaterials contact mode*: Direct contact, +; presence of connective tissue between the bone and biomaterials, −.

### 2.5. Statistical Analysis

The statistical analyses were performed with the aid of SPSS software for windows. Paired *t*-test was used for comparing the difference between the variables on right and left tibias and one-way analysis for comparing the variables with respect to separate days. Analysis of variance (ANOVA) was used for comparing the difference between groups with respect to separate days and post hoc analysis was used for comparing the variables two by two. The Wilcoxon signed-rank test was used for comparing the rate of inflammation between groups and on each group with respect to separate days. *P* < 0.05 was considered significant.

## 3. Results

The current study was performed on 32 rats (adult male Wistar rats aged 6–8 weeks). Two holes with a depth of 5 mm were created with a drill handpiece on the right and left tibias. Then the material was placed on the right side and the other cavity was left empty. After 7, 14, 28, and 60 days, histological, histomorphometrical, and radiographical evaluations were performed on samples obtained from the cavities. Radiographic density, inflammation rate, blood vessel count, bone lacunae density, bone cell count, trabecular bone thickness, and new bone formation amounts for each sample are shown in Table 1.

**Figure 2:**
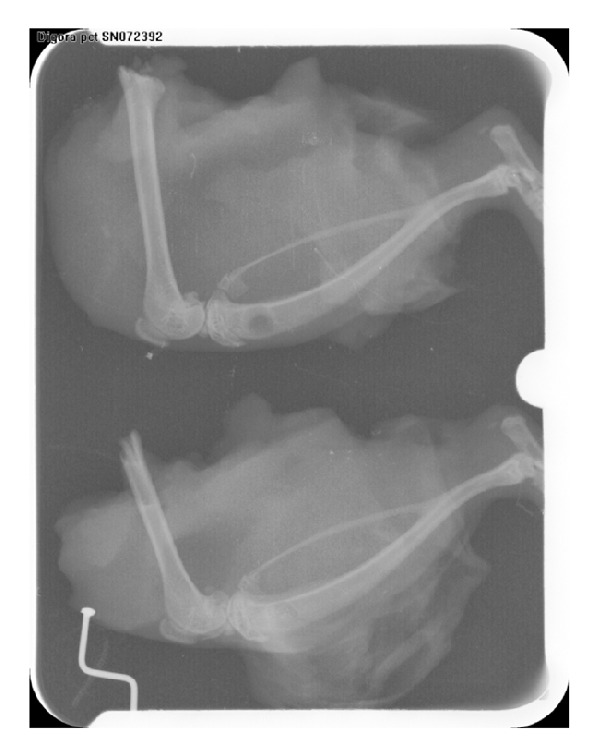
Radiographic view on day 7. A: Left tibia. B: Right tibia.

**Figure 3:**
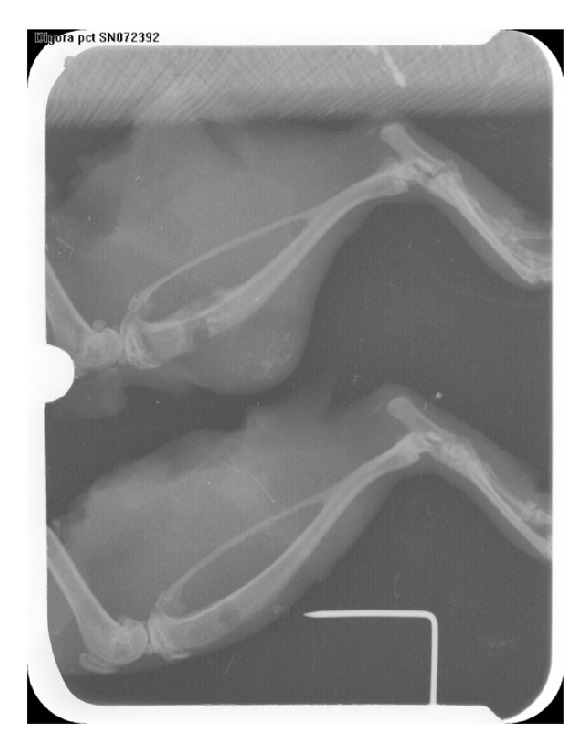
Radiographic view on day 14. A: Right tibia. B: Left tibia.

In accordance with histological findings on inflammation as shown in Table 2, the results were as follows. In the right tibias, 4 cases (12.5%) were Grade 0, 8 cases (25%) were Grade I, 2 cases (31.25%) were Grade II, 8 cases (25%) were Grade III, and 10 cases were Grade IV. In the left tibias, 6 cases (18.75%) were Grade 0, 6 cases (18.75%) were Grade I, 3 cases (6.25%) were Grade II, 8 cases (25%) were Grade III, and 9 cases (28.175%) were Grade IV. Foreign body reaction was not observed in any sample. In all cases the bones were vital and the bone-biomaterials contact mode in all cases was direct. According to histomorphometrical findings of trabecular bone thickness, the results were as follows. Throughout the whole study period, the maximum thickness of the bone trabeculae in the right tibias was 69.29 *μ*m and the minimum was 6.95 *μ*m. The maximum thickness of the bone trabeculae in the left tibias was 62.37 *μ*m and the minimum was 6.29 *μ*m. In right tibias, 3 cases (9.375%) were Grade I, 22 cases (68.75%) were Grade II, and 7 cases (18.75%) were Grade III. Of note is the fact that one rat was removed from the radiographic evaluation because of broken legs. The rate of radiographic density in the right tibias had a decreasing trend, and in the left tibias it decreased until day 14. Then it had an increasing trend, and the trends in right and left tibias are statistically significant (*P* values = 0.001, 0.013). Comparing the results obtained from digital densitometry indicated that the differences between the left and right tibias were significant at days 7 and 14 (*P* values = 0.035, 0.033) (Figures 2 and 3), and the right tibias had more radiographic density. Days 28 and 60 showed no significant differences (*P* values = 0.358, 0.223) (Figure 6). Presence of the material in the area of its application on day 7 caused more opacity than the control side due to rapidly breaking down nanoparticles and replacing them with bone which had less opacity than the material.

Inflammation on both sides declined over time, and the decreasing trend was statistically significant in right tibia defects (*P* value = 0.000). The number of blood vessels on both sides declined over time, but only on the left side there was a statistically significant decreasing trend (*P* value = 0.025). From day 7 to day 14, bone lacunae density increased, and then it decreased until day 60. The changes from day 7 to day 60 showed significant differences only in the left tibias (control) (*P* value = 0.024). The bone cell counts for the test and control side had decreasing trends. This difference is significant on both sides (*P* values = 0.001, 0.008, resp.), but between the experimental and the control the difference was not significant (*P* value = 0.586). In the test and the control groups, the increase in the thickness of the bone trabeculae was visible from day 7 to day 28. This trend continued until day 60 in the experiment side, but from day 28 to day 60 in the control side, the decrease in the average thickness of bone trabeculae was seen. The difference between the thickness of the bone trabeculae in the experimental defect and the thickness of the bone trabeculae in the control defect was not statistically significant with respect to separate days (*P* values = 0.832, 0.198, 0.295, and 0.236). However, the thickness of the bone trabeculae was more in the experimental defect than in the control defect on each day. The trend for new bone formation amount changes decreased from day 7 to day 14 and then increased until day 60, but this difference was significant only in the test group (*P* value = 0.034).

## 4. Discussion

In the present study, the tibia bone cavities of 32 rats were filled with injectable glass-ceramic-chitosan nanocomposite, and histopathological, histomorphometrical, and radiographical assessments were performed to evaluate its nanocomposite ability to regenerate rat bone. The results of the digital densitometer comparison indicated significant differences between the left and right tibias on days 7 and 14, but on days 28 and 60, the difference was not statistically significant. This may be due to the higher radio opacity of glass-ceramic-chitosan nanocomposite than bone and the uptake of nanoparticles during the first two weeks.

**Figure 4:**
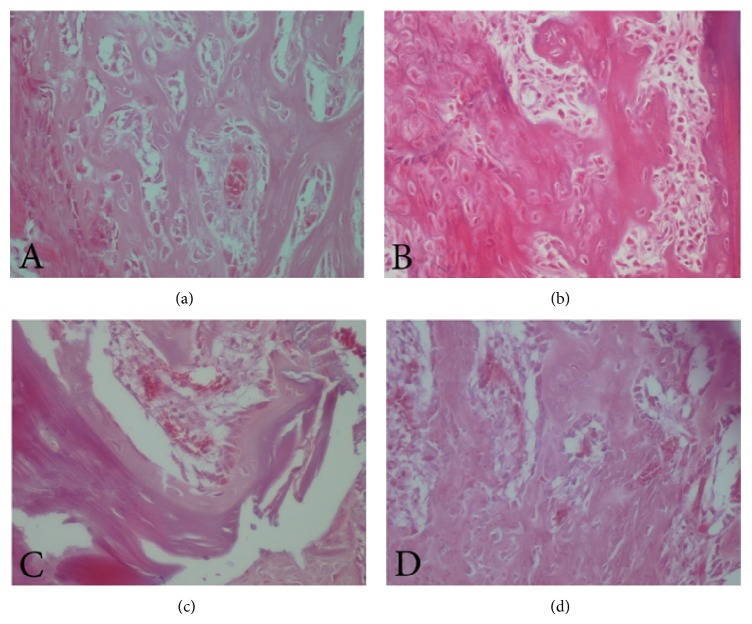
(a, b) Bone trabeculae on left and right tibias and (c, d) inflammation on left and right tibias on day 7.

**Figure 5:**
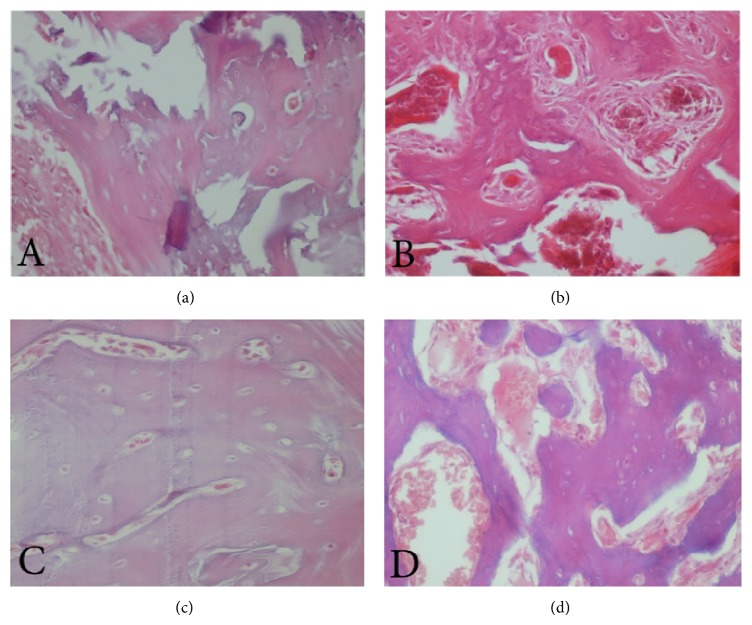
(a, b) Bone trabeculae on left and right tibias and (c, d) inflammation on left and right tibias on day 60.

**Figure 6:**
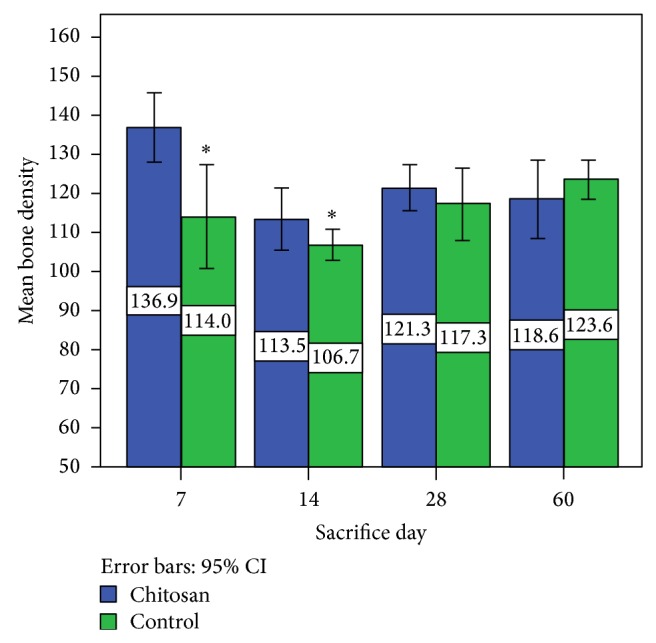
Mean bone density on different days of study.

The inflammation rate decreased over time in both the test and the control groups, but the differences were not significant (Figures 4(c), 4(d), 5(c), and 5(d)). The decreasing process was not statistically significant in the test groups, but significant progress in reducing inflammation was seen in the controls. There was no significant difference between left and right tibias regarding inflammation, perhaps because of the biocompatibility of glass-ceramic-chitosan nanocomposites and the lack of inflammation as a result. The biocompatibility of the mentioned material has been shown in a previous study on this material [14].

The number of blood vessels on both sides declined over time. This decreasing process was not statistically significant in the test groups, but significant progress in reducing the number of blood vessels was seen in the control group. There were no significant differences between the two sides at any period of the experiment. This decreasing trend represents the tissue repair process. During the second week after the wound was made, increased vascular system decreased [15].

Bone lacunae density containing osteocyte increased from day 7 to day 14 due to woven or immature bone formation. From day 14 to day 60, there was a decrease in the density of bone lacunae because of bone maturity and mature or lamellar bone formation. This trend was statistically significant only in the controls. However, the difference between experimental and control sides was not statistically significant.

The bone cell count increased from day 7 to day 14. The cause of this increase is osteoblasts activity and bone formation. From day 14 to day 60 bone cell counts decreased. The cause of this decline can be attributed to bone remodeling [16]. This trend was statistically significant in both the right and the left tibias, but between the cases and the controls, there was no significant difference in this respect.

The difference between the right and left tibias trabeculae thickness was near significant and indicated further trabeculae thickness in the placement area of the glass-ceramic-chitosan nanocomposites (Figures 4(a), 4(b), 5(a), and 5(b)). The percentage of bone formation was slightly less in right tibias than in left ones, although this difference was not significant. There was an increase until day 28, and then a decrease was seen in the controls.

The material that can be used to replace bone should be biodegradable, biocompatible, and effective. It should also be cost-effective and easy to use. Autogenic and allogenic grafts are popular methods for bone reconstruction [17]; however, using them is associated with problems such as infection, pain, restriction on harvesting and bone gain, duration of surgery, and the risk of death through tissue rejection [17, 18]. Unlike allogeneic and autologous bone grafts, xenogeneic grafts are widely available and do not require the patient to undergo additional surgery [18].

Injectable systems are highly regarded due to their minimal invasiveness [9]. Injectable systems used in the application of tissue engineering scaffolds can take the shape of the bone defect creating scaffolds in place and eliminating the need for premade implants [10]. Since these materials form themselves like the defect they fill, they can also be used in all places, even those with irregular shapes. The problem of cell adhesion and release of bioactive molecules through mixing before injection is also resolved [11, 12].

Several studies have evaluated the properties of chitosan in bone repair and have presented theories about its mechanism. Among them is a study by Park et al., in which the researchers observed that chitosan sponges enhanced the contact with bone and bone cell proliferation compared to controlled, polystyrene dishes. The researchers theorized that chitosan increased osteoblastic activity and osteogenesis [19]. According to results of the current study, a percentage of bone formation was slightly less in right tibias than in left ones, although the difference was not significant. Bone cells were more in the test group than in the control group, although the difference was not statistically significant. The difference of trabecular bone thickness between right and left tibias was near significant.

Daculsi et al. evaluated the biocompatibility and workability of injectable bone substitute in the form of implants in rabbit skeletal and nonskeletal tissues and observed that although this material had weak mechanical properties, it degraded fast and was replaced with new bone. It can be used in orthopedic and maxillofacial surgeries and also in routine clinical processes like pulp capping and root filling [17]. According to the current study, when glass-ceramic-chitosan nanocomposite was removed and replaced with bone after day 14, the radiographic density decreased significantly.

Weiss et al. clinically assessed an injectable bone substitute. Its effectiveness was analyzed for filling human dental sockets and preventing alveolar bone recession. Radiographic density measurement of the surgical defects showed an increase in bone formation rate. Meanwhile, the mentioned material's granules were in direct contact with bone, supporting bone growth. Gradual replacement of bone also led to alveolar bone crest height [20]. Results of the current study also demonstrated the following: the direct contact between bone and biomaterial, fast degradation of its nanoparticles, and no remaining material after day 14.

Seyedmajidi et al. evaluated the connective tissue reaction in recommended times with respect to the biocompatibility of injectable glass-ceramic-chitosan nanocomposite. They observed no significant difference regarding inflammation rate and blood vessel count between test and control groups after putting the material in polyethylene tubes under the rats' skin in different days. They also reported a decrease in inflammation rate during the study time. From their results, they realized the biocompatibility of this material [14]. Results of the current study also presented little inflammation and blood vessel rates and no foreign body reaction, thus showing the biocompatibility of glass-ceramic-chitosan nanocomposite.

In their study, Chevrier et al. theorized that chitosan increases angiogenesis in cartilage tissue on day 14 [21]. In the current study, a decrease in the blood vessel count was not observed in test group, although the blood vessel count of the controls declined in the study period.

Since we did not find any similar study using injectable glass-ceramic-chitosan nanocomposite regarding bone defect reconstruction, we could not compare the results in all respects regarding its workability.

## 5. Conclusion

Glass-ceramic-chitosan nanocomposite made no statistically significant difference regarding angiogenesis and new bone formation, but because of its biocompatibility, fast degradation and replacement of the material with bone, increased bone trabeculae thickness in surgery sites, and the test group's minimal difference with the control group, it can be used to reconstruct defects caused by traumas, tumors, infections, biochemical disorders, and skeletal malformations.

## Figures and Tables

**Table 1:** Radiographic density, inflammation rate, blood vessel count, bone lacunae density, bone cell count, trabecular bone thickness, and new bone formation amounts for each sample.

The variable studied	Rats number	Mean ± SD	Mean ± SD	*P* value
		Left	Right	
Radiographic density	31	115.4139 ± 11.01717	122.1065 ± 12.62336	0.030^*^
Blood vessel count	32	3.0625 ± 3.60946	2.3125 ± 1.80389	0.262
Inflammation rate	32	2.2500 ± 1.52400	2.3750 ± 1.47561	0.625
Bone lacunae density	32	67.1875 ± 40.45024	66.8125 ± 35.54782	0.931
Bone cells count	32	92.1563 ± 62.74532	95.0938 ± 54.00858	0.586
Trabecular bone thickness	32	29.0878 ± 13.36077	34.9725 ± 15.38281	0.058
New bone formation amounts	32	64.1719 ± 20.04381	62.1856 ± 20.7111	0.593

^*^ *P* value < 0.05 is statistically significant.

**Table 2:** Inflammation rate.

Day	Inflammation			
	*N*	Left	Right	*P* value
		Mean ± SD	Mean ± SD	
7	8	4.0000 ± 0.00000	3.2500 ± 1.38873	0.170
14	8	2.1250 ± 0.83452	2.3750 ± 1.06066	0.456
28	8	2.2500 ± 1.38873	2.5000 ± 1.30931	0.626
60	8	0.6250 ± 1.06066	1.3750 ± 1.68502	0.222
		*P* = 0.080	*P* = 0.000^*^	

^*^ *P* value < 0.05 is statistically significant.
